# Scapular morphometrics inform anatomic landmark distances for arthroscopic suprascapular nerve decompression: a cadaveric study

**DOI:** 10.1016/j.jseint.2026.101647

**Published:** 2026-01-29

**Authors:** Dave Osinachukwu Duru, Salma Chaudhury, Niel Kang, Cecilia Brassett

**Affiliations:** a Human Anatomy Centre, Department of Physiology, Development and Neuroscience, University of Cambridge, Cambridge, UK; b School of Clinical Medicine, University of Cambridge, Cambridge, UK; c Department of Trauma and Orthopaedics, Cambridge University Hospitals NHS Foundation Trust, Cambridge, UK

**Keywords:** Arthroscopic suprascapular nerve decompression, Superior transverse scapular ligament (STSL), Suprascapular nerve (SSN), Suprascapular artery (SSA), Scapular dimensions, Landmarks, Patient-specific, Cadaveric

## Abstract

**Background:**

Arthroscopic suprascapular nerve (SSN) decompression is an increasingly adopted treatment for SSN entrapment. It involves lateral-to-medial dissection to reach the suprascapular notch, placing adjacent neurovasculature at risk. Despite this, anatomical guidelines for safe dissection in this complex region remain limited. This study aimed to provide anatomical guidance for patient-specific arthroscopic SSN decompression.

**Methods:**

Twenty cadaveric shoulders from 10 cadavers were dissected. Distances from SSN and suprascapular artery (SSA) to the lateral insertion of the superior transverse scapular ligament (STSL) were measured. Distances from the lateral STSL to lateral acromion and acromioclavicular (AC) joint were recorded and correlated with measured scapular dimensions (height, spine length, and width).

**Results:**

The SSN and SSA were 7.0 ± 2.7 mm (range: 2.6-11.8 mm) and 4.2 ± 2.5 mm (1.4-9.5 mm) medial to the lateral STSL, respectively. The lateral STSL was 7.7 ± 1.0 cm (6.2-9.2 cm) from the lateral acromion and 4.5 ± 0.6 cm (3.1-5.5 cm) from AC joint, both distances significantly correlating with scapular dimensions (r = 0.45-0.77; *P* < .001 to *P* = .047).

**Conclusion:**

The SSA and SSN may lie as close as 1.4 mm and 2.6 mm from the lateral STSL, defining a “lateral danger zone” at the suprascapular notch. The data suggest blunt dissection may cautiously be performed up to 3.0 cm medial to the AC joint and 6.0 cm medial to the lateral acromion. Factoring individual scapular dimensions may further refine patient-specific operative reference distances. These findings may enhance pre-operative planning, surgical training, and intraoperative safety.

Suprascapular nerve (SSN) entrapment accounts for up to 2% of shoulder pain cases, but is likely underdiagnosed due to its subtle presentation and overlap with rotator cuff pathology.^1^^,^^5^^,^^14^^,^^15^^,^^25^ In overhead athletes, such as volleyball players, the prevalence reaches up to 33%, highlighting its significance in specific populations.^9^

The SSN provides motor innervation to supraspinatus and infraspinatus, and sensory fibers to the acromioclavicular (AC) and glenohumeral joints.^4^^,^^23^ Entrapment typically occurs at the suprascapular notch, where SSN courses beneath the superior transverse scapular ligament (STSL); this can result in progressive cuff atrophy, weakness, and posterolateral shoulder pain.^1^^,^^5^

SSN decompression, involving open or arthroscopic release of the STSL near its lateral insertion at the coracoid base, can restore function and relieve pain.^19^ In the past 2 decades, arthroscopic SSN decompression has gained popularity due to its minimally invasive nature, lower morbidity, faster recovery, and ability to address concurrent intra-articular pathology.^2^^,^^12^^,^^13^

However, the procedure remains technically demanding, with a steep learning curve.^24^ The SSN lies deep within the suprascapular fossa, close to the suprascapular artery (SSA). This results in a confined operative corridor and increases the risk of iatrogenic injury during arthroscopic decompression.^17^ Despite growing adoption and a range of described techniques, anatomical guidelines for safe arthroscopic access are lacking. Cadaveric studies report distances from the SSN to landmarks, such as the lateral aspect of the acromion or AC joint, to guide portal placement and dissection trajectory.^3^^,^^6^^,^^8^^,^^11^^,^^18^^,^^21^ While prior studies have reported landmark distances and examined associations with patient characteristics such as height and sex,^8^^,^^11^^,^^21^ they have not investigated operative distances in relation to scapular dimensions.

This cadaveric study aimed to (1) quantify the proximity of the SSN and SSA to the lateral STSL insertion at the coracoid base; (2) measure distances from the lateral acromion and AC joint to this insertion; and (3) evaluate correlations between these distances and scapular dimensions.

By identifying a neurovascular danger zone and quantifying distances from identifiable lateral landmarks to the suprascapular notch, this study provides anatomical context that may support portal planning, intraoperative orientation, and depth control during lateral-to-medial dissection. Rather than replacing direct arthroscopic visualization, these landmark distances are intended to complement it by suggesting risk-informed boundaries.

## Materials and methods

### Specimen preparation

Ten pairs of cadaveric shoulder specimens (n = 20) were provided by the Human Anatomy Centre within the Department of Physiology, Development and Neuroscience, University of Cambridge, UK. The donors (5 males and 5 females) had provided consent for anatomical research prior to death in compliance with the Human Tissue Act (2004). They were of White British descent and had a mean age at time of death of 80.8 ± 8.9 years. The donors were preserved by cannulation of the common carotid artery or femoral artery, and pressurized injection of a solution containing 38% ethanol, 1.5% methanol, 4.2% formaldehyde, and 56.3% distilled water. None of the donors had undergone previous shoulder surgery or had any recorded shoulder pathologies.

### Measurements

**Figure 1:**
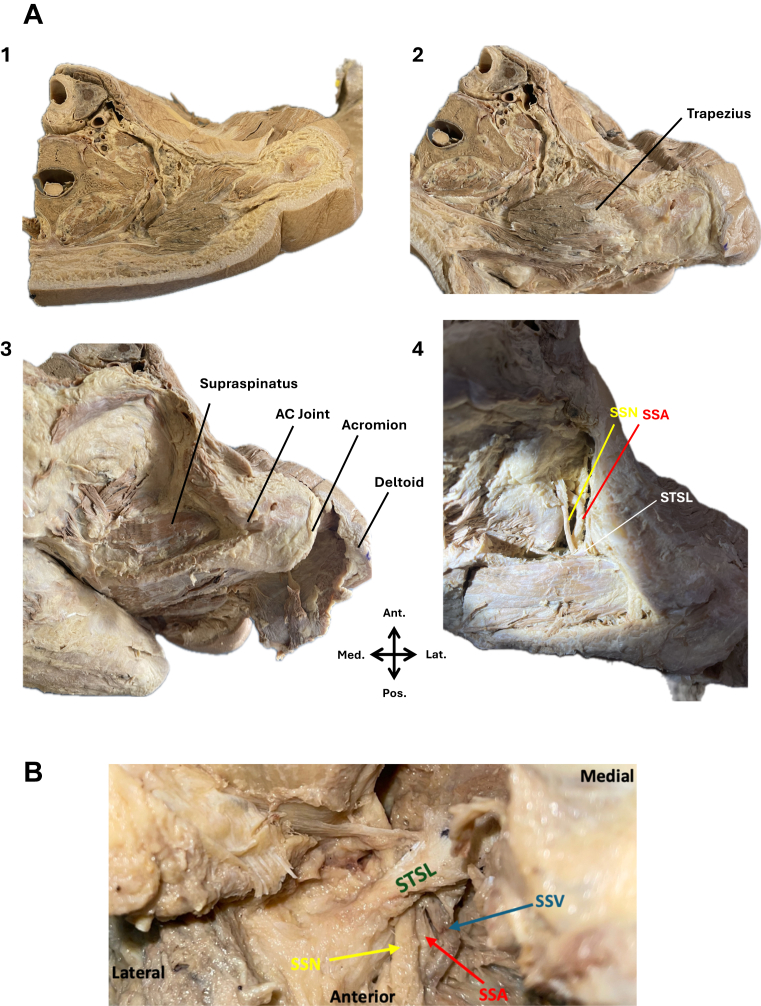
(**A**) Superior view of the right shoulder at various stages of dissection. (1) After removal of skin and subcutaneous tissue over the superior shoulder. (2) After removal of skin and subcutaneous tissue overlying the clavicle, acromion and scapular spine. (3) After reflecting trapezius to expose supraspinatus and reflecting deltoid to expose the lateral acromion. (4) After exposing the suprascapular nerve (SSN) coursing under the superior transverse scapular ligament (STSL). (**B**) The suprascapular notch. The suprascapular nerve (SSN), suprascapular artery (SSA), and suprascapular vein (SSV), all coursing under the STSL. In this specimen, both artery and vein coursed under STSL, a less common anatomical variation relevant to surgical orientation.

Dissection was performed via an open approach to expose the STSL, SSN, and SSA at the suprascapular notch (Fig. 1). Skin and subcutaneous tissue over the superior and posterior shoulder were removed. Trapezius was incised along its insertions on the clavicle, scapular spine, and acromion, and reflected laterally to expose the underlying supraspinatus. Deltoid was incised along its insertion on the scapular spine and acromion and reflected anterolaterally to expose the acromion. Supraspinatus was incised medially, 3 cm from the scapular border, and reflected posterolaterally from the supraspinous fossa. Fine dissection was then used to clear soft tissue, allowing visualization of the SSN, SSA, and the STSL at the suprascapular notch.

**Figure 2:**
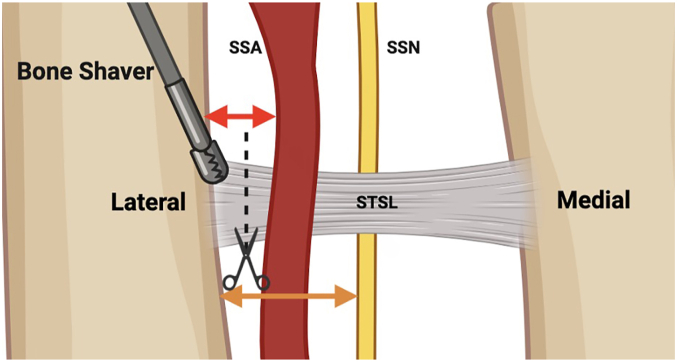
Zoomed-in superior view of the suprascapular notch region. This schematic diagram shows the typical site where the STSL is cut (indicated by scissors and *dashed lines*) and the lateral notch is shaved (indicated by the bone shaver). Note the proximity of the suprascapular artery (SSA) and suprascapular nerve (SSN) to the site of STSL release and bone shaving near the lateral insertion, indicated by the *red* and *orange arrows*, respectively. The suprascapular vein is excluded for illustrative purposes. *STSL*, superior transverse scapular ligament. [Figure made with BioRender].

**Figure 3:**
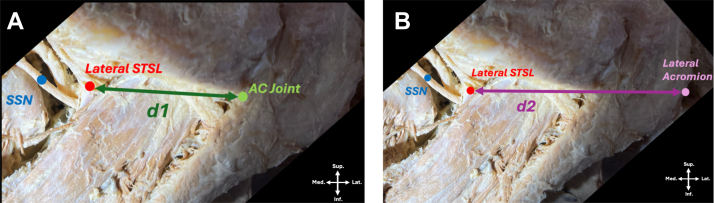
Relevant distances from external landmarks in arthroscopic suprascapular decompression. Superior view of dissected right shoulder, with trapezius removed to reveal bony landmarks and supraspinatus are exposed; both suprascapular artery (lateral) and suprascapular nerve (SSN) course under STSL. (**A**) = distance from the acromioclavicular (AC) joint to the lateral insertion of the STSL (d1). (**B**) = distance from the lateral acromion to the lateral insertion of the STSL (d2). *STSL*, superior transverse scapular ligament.

**Figure 4:**
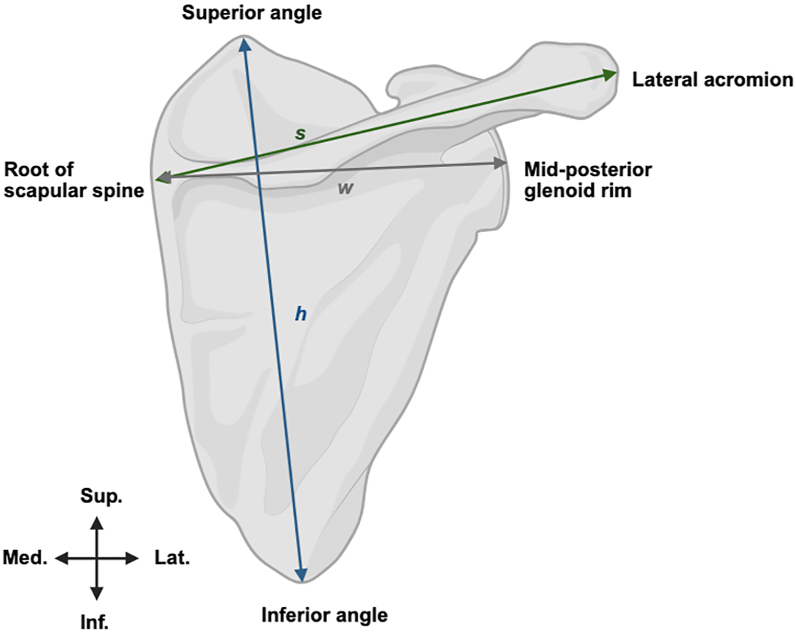
Measurement of scapular dimensions, Schematic figure of the posterior view of a *Right* scapula. Height (h) = distance from the superior angle to inferior angle. Spine length (s) = distance from the root of the scapular spine to lateral acromion. Width (w) = distance from the root of the scapular spine to the midposterior glenoid rim. *Sup.*, superior; *Inf.*, inferior; *Med.*, medial; *Lat.*, lateral. [Figure made with BioRender].

To quantify injury risk to the nerve and artery in SSN decompression, distances from the nerve and artery to the lateral STSL insertion at the coracoid base were measured (Fig. 2). Distances (mm) from the posterior aspect of the AC joint and lateral acromion (in line with posterior aspect of the clavicle) to the lateral STSL insertion were measured (Fig. 3). Scapular dimensions (height, spine length, and width) were measured (Fig. 4). All measurements were initially acquired to 0.1 mm using digital calipers and ImageJ software; scapular dimensions and distances from lateral landmarks to STSL were converted to centimeters and reported to 1 decimal place for clinical relevance. To ensure reliability, measurements were performed in triplicate by a single observer and verified by an independent observer, with inter-observer correlation >0.95.

### Statistical analysis

Statistical analyses were conducted using Prism v10.4.2 (GraphPad, San Diego, CA, USA) and Microsoft Excel v16.95.4 (Microsoft Corp., Redmond, WA, USA) Sample size is n = 20 throughout. The n-value represents the number of specimens involved in the analyses. To test for normal distribution, the Shapiro-Wilk test^20^ and Q-Q plots were employed. Simple linear regression was used to test for correlation between variables. Correlation strength was assessed using Pearson's r and corresponding R^2^ values. The F-test was used to investigate for statistically significant, nonzero relationships between variables. Statistical significance was attributed to differences for *P* values ≤.05.

## Results

The SSN and SSA were 7.0 ± 2.7 mm (2.6–11.8 mm) and 4.2 ± 2.5 mm (1.4–9.5 mm) medial to the lateral STSL insertion, respectively. There was no significant difference between left and right shoulders from the same donors (two-tailed paired *t*-test, *P* > .05). The SSA lay significantly closer to the lateral insertion of the STSL than the SSN (two-tailed paired *t*-test; *P* = .0024). Thus, the artery may be at greater risk than the nerve in release of STSL and burring in SSN decompression.

The mean distance from the lateral acromion to lateral STSL was 7.7 ± 1.0 cm (6.2–9.2 cm). The mean distance from the AC joint to the lateral STSL was 4.5 ± 0.6 (3.1–5.5 cm). The mean distance from the lateral acromion to the AC joint was 3.4 ± 0.8 cm (2.3–5.2 cm).

Distances from landmarks to the lateral STSL significantly correlated with measured scapular dimensions: height, spine length, and width (Table I). Scapular width was the best predictor of the distance from lateral acromion (r = 0.65; *P =* .0021) and AC joint (r = 0.77; *P <* .0001) to the lateral STSL, indicated by the highest R^2^ and lowest *P* values (Table II). This supports use of scapular dimensions as scaling parameters for landmark-based operative reference distances.

**Table I:** Measurement of scapular dimensions.

Scapular dimensions	All specimens	Left side	Right side
Height	14.6 ± 1.5 (11.1-16.7)	14.8 ± 1.6	14.4 ± 1.5
Spine length	14.0 ± 1.4 (11.4-16.1)	13.8 ± 1.6	14.3 ± 1.2
Width	10.8 ± 1.2 (8.5-13.0)	10.7 ± 1.2	10.9 ± 1.3

*SD*, standard deviation.

All measurements are in cm (mean ± SD). The range of measurements between the different shoulder specimens is indicated in brackets.

**Table II:** Correlations between scapular dimensions and study measures.

Graph	Distance from landmark to STSL vs. scapular dimension	Line of best fit equation	R^2^	*P* value
A	LA–STSL vs. height	Y = 0.2926∗X + 34.27	0.2017	.0470
B	LA–STSL vs. spine length	Y = 0.4276∗X + 16.93	0.3821	.0037
C	LA–STSL vs. width	Y = 0.5148∗X + 21.46	0.4182	.0021
D	AC–STSL vs. height	Y = 0.2298∗X + 11.77	0.2859	.0151
E	AC–STSL vs. spine length	Y = 0.3151∗X + 1.064	0.4769	.0007
F	AC–STSL vs. width	Y = 0.4069∗X + 1.442	0.6003	<.0001

*LA—STSL*, distance from the lateral acromion to the lateral insertion of the superior transverse scapular ligament; *AC—STSL*; distance from the acromioclavicular joint to the lateral insertion of the superior transverse scapular ligament.

LA–STSL vs. scapular dimension: scatter plot assessing relationship between distances from the lateral acromion to lateral STSL insertion vs. scapular height/spine length/width; AC–STSL vs. scapular dimension: scatter plot assessing relationship between distances from the acromioclavicular joint to lateral STSL insertion vs. scapular height/spine length/width. Significance indicated by *P* ≤ .05.

## Discussion

Despite prior studies suggesting risk of neurovascular injury during arthroscopic SSN decompression,^18^ this study defines a quantitative “lateral danger zone.” This zone defines a high-risk area in surgical release of the STSL. The SSA and SSN lie dangerously close to the site of STSL release near the lateral insertion, with minima of just 1.4 mm and 2.6 mm medial to this insertion. These measures can enable surgeons to identify and protect the neurovasculature intraoperatively during dissection, burring, and STSL release. This is clinically important, as iatrogenic injury can result in neuropathy or excessive bleeding, potentially obscuring the arthroscope and surgeon's view.^7^^,^^16^

Based on minimum distances from the AC joint and lateral acromion, landmarks useful for arthroscopic portal placement, to the STSL, our data suggest the upper bounds for cautious dissection are 3.0 cm medial to the AC joint and 6.0 cm medial to the lateral acromion. This guidance supports the anteromedial dissection used from lateral and posterior portals toward the coracoid base in arthroscopic SSN decompression.^2^^,^^3^ However, the substantial inter-individual variation in distances to the lateral STSL (range: 3.1-5.5 cm from the AC joint and 6.2–9.2 cm from the lateral acromion) suggests that reliance on fixed measures is insufficient for surgical guidance. We established that distance measures correlate significantly with scapular dimensions. These correlations enable surgeons to adjust dissection margins according to individual scapular morphology, based on input into the derived line of best fit equations (Table II). Scapular dimensions can be estimated pre-operatively using surface palpation or imaging modalities such as computed tomography (CT); however, surface palpation provides an external approximation influenced by the soft tissue envelope, whereas imaging reflects underlying bony anatomy. Thus, the 2 are not directly interchangeable. Although conventional CT may underestimate true anatomic distances, three-dimensioanl CT correlates closely with anatomic measurements and may provide superior accuracy when available.^8^ Nevertheless, these measures, such as scapular spine length, could be incorporated into pre-operative protocols for arthroscopic SSN decompression, facilitating patient-specific guidance. For example, patients with smaller scapular spine lengths, measured clinically or radiographically, would be expected to have proportionally shorter distances from landmarks to the suprascapular notch, prompting more conservative medial dissection limits. Conversely, larger scapular dimensions would permit proportionally greater safe dissection margins. In this way, scapular morphometrics allow scaling of risk-informed operative boundaries, rather than reliance on fixed guides.

The landmark relations from the lateral acromion and AC joint to the STSL were previously investigated by Knudsen et al.^11^ Their measured values differed slightly from the present study. The mean distance from the lateral acromion to the suprascapular notch was 7.7 ± 1.0 cm in the present study but 6.6 ± 0.5 cm in the study by Knudsen et al. They also reported that the AC joint was 3.6 ± 0.5 cm from the STSL, whereas the present study measured 4.5 ± 0.7 cm. Both measures from the acromion were done via open dissection, but Knudsen et al measured the AC relation arthroscopically, potentially explaining the 0.9 cm discrepancy. Dietrich et al,^8^ 2015 measured the distance from the anterolateral acromion to the STSL as 6.4 ± 0.6 cm via anatomic dissection but just 4.7 ± 0.5 cm via CT. Another CT-based study in a Japanese population reported a shorter mean distance of 4.3 ± 0.5 cm from the lateral acromion to the suprascapular notch, though without arthroscopic or open anatomic validation.^21^ These differences likely reflect variations in anatomical reference points, population morphology, small variable sample sizes, and measurement methods. For instance, CT measurements are often noted to underestimate anatomical measures.^8^ The variation in landmark distances underscores a need to mitigate surgical reliance on standardized measures and instead promote the use of patient-tailored anatomical frameworks.

Despite the strengths of the present study, the limitations must be addressed. The sample size is small, with just 10 unique individuals (20 shoulders). This limits the generalizability to wider populations. Next, the formalin fixation used for the cadavers may have shifted nerve and tissue positioning, potentially causing divergence from the living state.^22^ Fresh frozen specimens may have been used instead, as they better recapitulate the living state and may have enabled easier visualization of adipose tissue. Also, our elderly cadaveric population may not fully represent the younger, athletic demographic typically affected by SSN entrapment.^25^ However, the anatomical relationships we describe are preserved across ages, and our equal gender distribution provides balanced data. The open dissection approach, while necessary for accurate measurement, may not perfectly replicate arthroscopic perspectives.^10^ The absolute distances may differ *in vivo* due to patient positioning and arthroscopic fluid distension. Thus, the reported values should not be interpreted as permitting blind dissection; direct visualization of the suprascapular notch and neurovascular structures remains essential throughout arthroscopic decompression. Nevertheless, our baseline data of the relative anatomical relationships remains imperative and may support future studies.

Future research should validate the quantified “lateral danger zone,” as well as the anatomical relationships between scapular size and landmark relations, via three-dimensioanl CT scan-based and arthroscopic measurements in live patients. This could confirm anatomical accuracy from the surgeon's perspective. As donor height was not available for the present study, future studies can assess its potential correlations to scapular dimensions. Additionally, we recommend prospective studies integrating patient-specific scapular dimensions into pre-operative surgical planning for arthroscopic SSN decompression, with evaluation of the effect on safety, operating time, and patient outcomes. Investigation across diverse ethnic populations would enhance the global applicability of our findings.

## Conclusion

This study defines a quantitative “lateral danger zone” at the suprascapular notch and introduces morphometric predictors that inform patient-specific assessment of operative risk in the suprascapular notch region. By correlating surgical landmark relations with scapular dimensions, we provide anatomical guidance that can inform pre-operative planning, enhance intraoperative safety, and support surgical training. Incorporating these patient-specific measurements into arthroscopic SSN decompression may help reduce iatrogenic risk, improve precision, and facilitate broader adoption of this technically demanding surgical procedure.

## Disclaimers:

Funding: This work was funded in part by the Royal College of Surgeons of England Intercalated Bachelor of Science Degree in Surgery Award.

Conflicts of interest: Niel Kang reports payment or honoraria for lectures, speakers' bureaus, presentations, manuscript writing or educational events from Mathys, and support for attending meetings and/or travel from Arthrex, all of which are unrelated to this study. All the other authors, their immediate families, and any research foundations with which they are affiliated have not received any financial payments or other benefits from any commercial entity related to the subject of this article.

## Data availability

All data supporting the findings of this study are available within the paper. Additional data are available from the corresponding author upon reasonable request.
